# Recent advances of targeted therapy in relapsed/refractory acute myeloid leukemia

**DOI:** 10.17305/bjbms.2020.5485

**Published:** 2021-08

**Authors:** Jiale Ma, Zheng Ge

**Affiliations:** 1 Department of Hematology, Zhongda Hospital, School of Medicine, Southeast University, Institute of Hematology Southeast University, Nanjing, China; 2 Department of Hematology, Xuzhou Central Hospital, Xuzhou, China

**Keywords:** Relapsed/refractory acute myeloid leukemia, targeted therapy, novel genetic mutations, immunotherapy

## Abstract

Despite advances in the understanding of disease pathobiology, treatment for relapsed or refractory acute myeloid leukemia (R/R AML) remains challenging. The prognosis of R/R AML remains extremely poor despite chemotherapy and bone marrow transplants. Discoveries on recurrent and novel genetic mutations, such as FLT3-ITD and IDH1/IDH2, critical signaling pathways, and unique molecular markers expressed on the surface of leukemic cells have been under investigation for the management of R/R AML. Other than monoclonal antibodies, diabodies, and triabodies are new targeted therapies developed in recent years and will be the new direction of immunotherapy. Targeted agents combined intensive regimens can be viable options for salvage therapy and as bridges to allogeneic transplant. Future directions will focus on novel, efficient and targeted combinations, low-toxicity maintenance, and individualized precision strategies. Here, we review the major recent advances of targeted therapies in the treatment of R/R AML.

## INTRODUCTION

Acute myeloid leukemia (AML) is a clinically and biologically heterogeneous disease, characterized by clonal proliferation of myeloid precursors. Acquired somatic mutations accumulated in hematopoietic stem and progenitor cells are the main pathogenesis of AML [1]. Clinically, R/R AML remains the most challenge issue with an extremely poor prognosis. It is reported that the median overall survival (mOS) of patients with R/R AML from relapse was about 6 months, with a 5-year OS of 10% [2]. There is no universally accepted standard therapy for R/R AML other than the enrollment into clinical trials. Traditional therapeutic options include cytarabine-base salvage chemotherapy, HSCT, low-dose cytarabine or hypomethylating agents, and best supportive care (BSC) alone. The most commonly used salvage chemotherapy include FLAG-IDA (fludarabine, cytarabine, idarubicin, and granulocyte colony-stimulating factor) [3,4], CLAG (cladribine, cytarabine, and G-CSF) [5,6], and MEC (mitoxantrone, etoposide, and cytarabine). In patients fit for intensive chemotherapy, the complete remission (CR) rates range from 44% to 59.4% and the overall survival (OS) ranges from 6.2 to 8.7 months [7]. However, few patients with R/R AML are cured. Allogeneic HSCT is considered to be the only curative treatment in R/R AML patients, whereas only a minor proportion is able to proceed to allo-HSCT because of unfit or other factors. For younger patients with R/R AML, the best choice is the re-induction of CR by intensive chemotherapy, and followed by allo-HSCT. The outcome of older adults is even worse, likely due to limited tolerability for intensive chemotherapy, high-risk disease biology with adverse cytogenetic and molecular abnormalities, and the chemotherapy-resistant nature of blasts [8]. In patients unfit for intensive chemotherapy and allo-HSCT, hypomethylating agents (HMAs) have been present encouraging efficacy in unfit and older R/R patients. HMAs can induce an OR in 17%-26% of cases with a median survival of 6-9 months [9,10]. The results of phase III study have shown that azacitidine (AZA) maintenance after CR/CRi after intensive chemotherapy significantly improves Disease-Free Survival (DFS) [11]. In patients relapsing after allo-HSCT, HMAs are of benefit. A prospective trial in 39 MDS or AML patients relapsed within 100 days of transplantation, Aza showed an ORR of 30% and CR rate of only 7.7% [12]. Due to the poor results, it is urgently to seek novel therapies to improve the response rate.

The identification of unique molecular markers expressed on the surface of leukemic cells and discoveries on recurrent and novel genetic mutations are pivotal for the discovery of novel targeted therapies against R/R AML. Traditional chemotherapy acts not only on AML blasts but also on normal cells and produces toxicity. Targeted agents mainly aim at genetic or molecular lesions specific to, or enrich in, AML cells. This difference will make targeted therapy be more effective and less toxic than conventional chemotherapy. In recent years, the development and application of whole-genome sequencing have given us a macroscopic understanding of the AML gene mutation spectrum [13]. Based on these studies, small molecule targeted drugs had achieved remarkable results. Over the past few years, the Food and Drug Administration (FDA) approved many novel treatment options, including venetoclax in combination with HMAs or LDARAC [14] and glasdegib in combination with LDARAC [15], for the treatment of newly diagnosed or older patients to improve the clinical outcome. Some research results of targeted therapy have gradually enriched and changed the current clinical treatment plan for R/R AML.

## TARGETED GENES FOR R/R AML

### FLT3-ITD inhibitors

FMS-like tyrosine kinase 3 (FLT3) internal tandem duplication (ITD) mutations in patients with acute myeloid leukemia (AML) are associated with early relapse and poor prognosis. FLT3-ITD mutation can lead to constitutive autophosphorylation of FLT3 and activation of its downstream effectors including RAS/RAF/MEK, MAPK/ERK, PI3K/AKT, and JAK/STAT signal pathways, result in uncontrolled cell proliferation, survival, and differentiation of AML, while FLT3-ITD inhibitors can inhibit these downstream pathways through specific FLT3 inhibition [16]. The first-generation FLT3 inhibitors such as midostaurin (protein kinase C inhibitor), sunitinib (VEGFR inhibitor), sorafenib (RAF inhibitor), lestaurtinib, and ponatinib (BCR-ABL inhibitor) were multitargeted kinase inhibitors with short duration of response [17]. The second-generation of FLT3 inhibitors includes quizartinib, gilteritinib and crenolanib, and pexidartinib and was more selective and less off-target than the first generation. Of all these FLT3-ITD inhibitors, midostaurin and gilteritinib have been approved by FDA for FLT3 mutated AML.

Sorafenib. Sorafenib is a multi-targeted small molecule inhibitor of RAF kinase, VEGFR-2, c-KIT, and FLT3, with activity of down-regulation of the MAPK pathway, Mcl-1 (Myeloid cell leukemia-1), and growth inhibition of AML cells with FLT3-ITD mutations [18-20]. Sorafenib was first approved by the FDA for the treatment of renal cell cancer and hepatocellular carcinoma, but it also has been used in R/R AML with wide experience. Sorafenib monotherapy showed only modest clinical activity in R/R AML at multiple dose levels with CRc rates ranging from 0 to 11.1% [21,22]. However, 63 patients with R/R FLT3-ITD AML after allo-HSCT or conventional therapy were enrolled in a multi-center study, sorafenib monotherapy showed an ORR of 83% and CRc of 23%. About 47% of patients without prior allo-HSCT after a median treatment duration of 136 days developed sorafenib resistance, while allo-HSCT group showed lower (38%) and significantly later (197 days, P=0.03) sorafenib resistance conversely [23]. The long-term follow-up results of the allo-HSCT group showed that 6 of 29 patients (21%) are still alive with a median follow-up of 7.5 years and 17% achieved sustained complete remissions [24]. Sorafenib combination therapies have demonstrated activity in patients with R/R AML. However, responses are of limited duration. Combinations of sorafenib and DNA methyltransferase inhibitors (HMAs, decitabine [Dec], and azacitidine [Aza]) have been explored in three trials. In 37 R/R and untreated unfit AML patients, Aza plus sorafenib showed a CRc of 46% and mOS of 6.2 months [25]. In addition, sorafenib plus the Aza showed a CRc of 50% and mOS of 10.7 months in 8 patients with FLT3-ITD+ AML who had relapsed following allo-SCT [26]. Sorafenib plus Dec showed a CRc of 83% and mOS of 5.2 months in 6 FLT3-ITD R/R AML [27]. In a phase I/II study, combinations of sorafenib with idarubicin and Ara-C in 7 R/R FLT3-mut patients achieved a CRc of 43% [28]. Eighty-three relapsed AML patients were enrolled in a retrospective study to evaluate the efficacy of sorafenib combined with other therapeutic strategies for AML with FLT3-ITD relapsed after allo-HSCT. The CR and OR rates were higher in sorafenib cohort than the non-sorafenib cohort (66% vs. 30% of CR, *p* = 0.002 and 83% vs. 50% of OR, *p* = 0.001), so as the 1-year OS (46.8% vs. 20%, *p* = 0.003) and the 1-year PFS (44.9% vs. 16.7%, *p* = 0.001). Subgroup analysis showed that the CR and OR rates in sorafenib+ chemotherapy+donor lymphocyte infusion (DLI) were higher than that in monochemotherapy (*p* = 0.006, *p* = 0.001), and they were similar to that in sorafenib+ chemotherapy and chemotherapy +DLI (all *p* > 0.008) [29]. The combination of sorafenib, plerixafor (a SDF-1/CXCR4 inhibitor), and G-CSF to increase mobilization and elimination of FLT3-ITD progenitor cells was conducted in a phase I trial, and 28 patients with R/R FLT3-ITD-mutated AML were enrolled, showing an ORR of 37% [30].

Midostaurin. As another first-generation FLT3 inhibitor, midostaurin appeared transient monotherapy activity. However, a recent study showed that chemotherapy plus midostaurin led to improved outcomes of newly diagnosed AML for *FLT3* mutations. In this phase III trial, 3277 patients were enrolled to determine whether the addition of midostaurin to standard chemotherapy would prolong overall survival in untreated AML with *FLT3* mutations. The mOS was significantly longer in the midostaurin group (74.7 months) than in the placebo group (25.6 months, *p* = 0.009), as was event-free survival (8.2 vs. 3.0 months). The 4-year OS rate was 51.4% and 44.3%, respectively [31]. In R/R *FLT3*-mut AML patients, several clinical trials have been conducted (Table 1). A phase II trial explored midostaurin monotherapy in 95 R/R or newly diagnosed unfit AML patients, with the overall response rate (CR, PR, HI, BR) of 71% and 56% in FLT3-mutant group and FLT3 wild-type group, respectively [32]. As for midostaurin combinations, Williams et al. conducted a phase I trial to determine the clinical maximum tolerated dose (MTD), and recommended phase II dose of midostaurin combined Dec in newly diagnosed or R/R AML. In this study, 16 patients were enrolled, and the ORR was 36.4%. About 25% of patients achieved CR or Cri, and the median duration of remission was 107 days (range from 28 to 331 days) days [33]. 10 R/R AML patients received midostaurin, all-trans retinoic acid (ATRA) and CLAG (cladribine, Ara-C, G-CSF) chemotherapy in a phase I trial, achieving a CR rate of 22% and median OS of 3.5 months [34]. Another two trials explored the combination of midostaurin and AZA in R/R or untreated AML, the first one enrolled 54 R/R AML and untreated AML patients with a ORR of 26% [35], and the other one included 17 patients with a ORR of 18%, and the median OS was 6 months in the second study [36]. A phase I trial analyzed midostaurin and bortezomib with/without MEC (mitoxantrone, etoposide, and cytarabine) in 34 patients. A 56.5% CR rate and 82.5% ORR were observed. Of noted, MEC included cohort obtained the median OS of 11 months and a significant higher CR rate of 57% compared with the cohort without MEC (0%) [37]. Therefore, more novel combination therapies are worthy of further exploration.

**TABLE 1:**
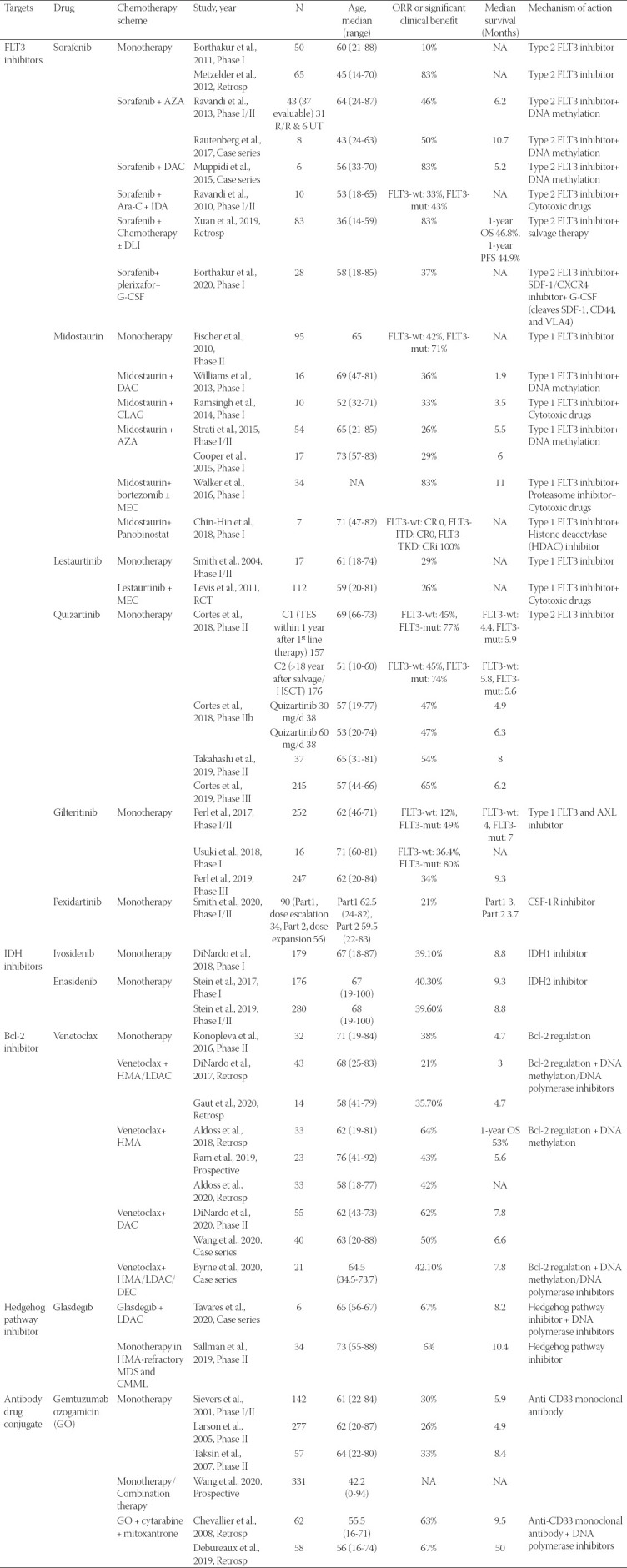
Results of targeted agents for R/R AML/MDS

Lestaurtinib. Lestaurtinib demonstrated no surprising results in R/R AML. A phase I/II open-label trial of lestaurtinib single-agent therapy in R/R or poor-risk AML showed an ORR of 29% [38]. The results form Cephalon 204 trial indicated that the addition of lestaurtinib to salvage chemotherapy provides no benefit to AML patients with FLT3 mutations in first relapse. Two hundred and twenty-four patients were enrolled in the randomized trial to received chemotherapy alone or followed by 80 mg of lestaurtinib twice daily. The total CR/CRp rate was 26% in lestaurtinib arm and 21% in the control group, respectively (*p* = 0.35), and there was no difference in overall survival between the two arms [39].

Quizartinib. Quizartinib was approved by the Ministry of Health, Labor and Welfare (MHLW) of Japan for R/R AML with FLT3 mutation. Compared with the first-generation TKIs, quizartinib has a stronger selectivity for FLT3. Monotherapy of quizartinib demonstrated a great efficacy in R/R AML, with a CR rate of 40-50%, and OS of 5-8 months [40-42]. A phase IIb study (NCT01565668) evaluated the efficacy and safety of 30 mg or 60 mg dosing regimens of quizartinib monotherapy in patients with R/R AML with FLT3 mutations. Of all 76 patients, CRc rates were 47% in both groups, similar to earlier reports with higher quizartinib doses. Incidence of QTcF above 480 ms was 11% and 17%, and QTcF above 500 ms was 5% and 3% in the 30 and 60 mg groups, respectively, which was less than earlier reports with higher doses of quizartinib. Median OS (20.9 vs. 27.3 weeks), duration of CRc (4.2 vs. 9.1 weeks), and bridge to transplant rates (32% vs. 42%) in 60 mg group were higher than 30 mg group. Quizartinib at an appropriate high dose may be more beneficial [40]. In addition, a phase II study of oral quizartinib in Japanese patients with FLT3-ITD positive R/R AML achieved a CRc rate of 53.8%. The median duration of CRc and OS was 16.1 weeks and 34.1 weeks, respectively. The major adverse events (AEs) were febrile neutropenia (43.2%), platelet count decreased (37.8%), and QT prolonged (35.1%) [41]. A phase III trial (QUANTUM-R trial) assessed single-agent quizartinib could improve overall survival versus salvage chemotherapy. Three hundred and sixty-seven patients were enrolled in this trial, of all patients, 245 were randomly allocated to quizartinib, and 122 to chemotherapy. The results showed that mOS was 6.2 months (5.3-7.2) in the quizartinib group and 4.7 months (4.0-5.5) in the chemotherapy group with median follow-up for 23.5 months (IQR 15.4-32.3). Hematological toxicity, pneumonia, and QT prolongation were the most common AEs, and no grade 4 events occurred. There were 33% treatment-emergent deaths in the quizartinib group (13% of which were due to AEs) and 17% in the chemotherapy group (10% of which were due to AEs) [42]. In the QUANTUM-R trial, single-agent quizartinib significantly improves the mOS from 4.7 months to 6.2 months compared with another phase III trial (DATAML study), while the rate of CR or CRi was lower than DATAML study (27% vs. 49%). The main characteristics of patients were similar between the two trials. However, 4.4% of patients received a low-intensity regimen as a salvage treatment in DATAML study, whereas about 25% of patients received LDAC treatment in the QUANTUM-R study. This maybe a major factor contribute to the difference in response rate and OS between two studies [43]. A recent study demonstrated that glucocorticoids (GCs) enhance the antileukemic activity of FLT3 inhibitors in FLT3-mutant acute myeloid leukemia. Gebru et al. found that treatment of FLT3 internal tandem duplication AML cells with quizartinib using RNA sequencing and drug screening had upregulated inflammatory genes in drug-tolerant “persisters” (DTPs) and therefore enhanced susceptibility to anti-inflammatory GCs. Combination of FLT3 inhibitors and GCs is promising in eliminate DTPs and prevents relapse in FLT3-mutant AML [44]. Although quizartinib displayed promising initial clinical trial results in previous studies, FDA rejected approval for quizartinib for R/R AML In June 2019. The reasons are listed as follows, first, the QuANTUM-R phase III results got a significant OS benefit, but the median survival was only extended 6 weeks (6.2 vs. 4.7 months) compared with chemotherapy group. In addition, there was no difference in event-free survival, which raised questions about what led to the improvement in OS [45]. Third, bias in provision of data and the number of transplants in each group raised questions as to whether this affected differences in survival between the two groups. Another concern was the unique toxicities of QTc prolongation and myelosuppression, but this did not appear to be a major factor for FDA rejection.

Gilteritinib. Gilteritinib is a small molecule dual inhibitor of FLT3/AXL [46]. Phases I/II studies in relapsed/refractory (R/R) AML with or without FLT3 mutations of gilteritinib established the daily dose of 120 mg gilteritinib for further clinical phase III trials. In these two clinical trials, gilteritinib was given as daily escalating doses. The ORR was 40%-80%. The most common adverse events were diarrhea, anemia, fatigue, and liver enzyme elevation [47,48]. In a phase III trial (ADMIRAL study), 371 R/R AML with *FLT3*-mutated were enrolled to compare the efficacy and survival of gilteritinib and chemotherapy. This study showed that gilteritinib resulted in significantly longer survival and higher percentages of patients with remission than salvage chemotherapy with OS 9.3 months vs. 5.6 months and median event-free survival 2.8 months vs. 0.7 months, respectively. The ORR was 34.0% in the gilteritinib group and 15.3% in the chemotherapy group, and CRR was 21.1% and 10.5%, respectively. The most common AEs of grade 3 or higher in the gilteritinib group were febrile neutropenia (45.9%), anemia (40.7%), and thrombocytopenia (22.8%), but all these AEs occurred less frequently in gilteritinib group than in chemotherapy group [49,50]. The combination of gilteritinib with Aza (NCT02752035), VEN (NCT03625505), and atezolizumab (NCT03730012) is being studied. Of note, the U.S. FDA label for gilteritinib indicates several significant warnings and precautions, including the risk of differentiation syndrome, posterior reversible encephalopathy syndrome (PRES), and prolongation of the corrected QT interval (QTc) [51]. In the ADMIRAL trial, a prolonged QTc interval occurred in 5% participants, with only 1 subject had an increase in the QTc to > 500 ms. Therefore, it is recommended to monitor electrolytes, potassium, and magnesium levels throughout gilteritinib therapy [52]. According to the package insert of gilteritinib, an ECG should be performed at baseline, the day 8 and 15 of cycle 1, and before the start of cycles 2 and 3. If the QTc interval increases over 500 ms, gilteritinib should be held. If the QTc improves less than 480 msec or within 30 ms of baseline, gilteritinib may be restarted at 80 mg/day. When the QTc increases by over 30 ms, if confirmed by repeat ECG, dose reduction should be considered. In the treatment of R/R AML co-infection, it is inevitable to use antifungal drugs. However, strong CYP3A inhibitors (such as voriconazole and posaconazole) have been shown to increase gilteritinib concentration and therefore have the potential to increase the risk of toxicity. Unless necessary, toxicity should be monitored more frequently. Grapefruit and its juice strongly inhibit CYP3A4 and should be avoided during gilteritinib therapy. In addition, P-gp and strong CYP3A inducers may decrease gilteritinib exposure, so the combination of gilteritinib and P-gp or strong CYP3A inducers is not recommended. Besides, gilteritinib has the potential to reduce the efficacy of drugs that target sigma non-specific receptor and/or 5HT2B, such as escitalopram, fluoxetine, or sertraline. Therefore, alternative medications are recommended unless these drugs are considered essential.

Pexidartinib. Pexidartinib is a selective small-molecule kinase inhibitor of CSF1R, KIT, and FLT3-ITD. A phase I/II study of pexidartinib monotherapy in R/R AML demonstrated an ORR of 21% and CRR of 11%, which was lower than the ~40% to 50% CRc rates observed with quizartinib [40,53] or gilteritinib [47]. Median OS of dose expansion group and responders with complete remission was 3.7 and 8.8 months, respectively [54].

### IDH inhibitors

Isocitrate dehydrogenase 1 and 2 (IDH1 and IDH2) mutations are recurrent mutations in AML. Somatic point mutations in IDH1/2 lead to the excessive secretion of D-2-hydroxyglutarate (D-2HG). D-2HG plays an important role in the development of both hematological and solid tumors. It mainly promotes the occurrence of tumors by interfering with cellular metabolism and epigenetic regulation, thus leading to the expansion and differentiation of hematopoietic stem cells [55,56]. In addition, D-2HG can detect IDH1/2 mutations at the time of diagnosis and can also predict clinical response [57]. Small molecule inhibitors targeting IDH1/2 have shown strong therapeutic activity in clinical studies.

Ivosidenib. Ivosidenib (AG-120) is an oral, targeted, small-molecule inhibitor of mutant IDH1. It can restore normal differentiation and results in clinical responses in a subset of patients with mIDH1 R/R AML. The outgrowth of RTK pathway mutations and 2-HG-restoring mutations contributed to acquire resistance [58]. In a previous phase 1 clinical trial, 179 R/R AML of all enrolled patients (258) received ivosidenib, 500 mg once daily. Monotherapy with enasidenib yielded an ORR of 39.1%, CR rate of 21.8%, and CR plus CR with partial hematologic recovery (CRh) rate of 30.2%. The mOS in the primary efficacy population was 8.8 months. The main treatment-related AEs were prolongation of the QT interval (in 7.8% of R/R AML patients), the IDH differentiation syndrome (in 3.9%), anemia (in 2.2%), thrombocytopenia or a decrease in the platelet count (in 3.4%), and leukocytosis (in 1.7%). Thus, monotherapy with enasidenib was overall well tolerated [59]. However, the CR+CRh and CR rates appear higher in the mIDH1 newly diagnosed population compared with the mIDH1 R/R AML population in this and Roboz’s study. In patients with newly diagnosed *mIDH1* AML, single agent treated with ivosidenib achieved a CR/CRh rate of 42.4% and mOS of 12.6 months [60].

Enasidenib. Enasidenib (AG-221) is an oral small-molecule IDH2 inhibitor that is approved by FDA in 2017 for treatment of adult patients with mutant-IDH2 R/R AML at an initial dose of 100 mg once daily. The phase I/II study of enasidenib induced overall responses in 40.3% of patients with R/R AML, with 19.3% of patients achieving CR. The mOS was 9.3 months, and 19.7 months in those with CR. Hyperbilirubinemia and IDH-differentiation syndrome (IDH-DS) were the most prominent toxicities [61]. In a dose-escalation and expansion trial, 214 of 345 AML patients (62%) with R/R AML received enasidenib, 100 mg/d. Monotherapy with enasidenib yielded a CR rate of 19.6%, 10.3% patients proceeded to an allo-HSCT, and the ORR was 38.8%. MOS was 8.8 months. Similar results of ORR were demonstrated among patients who were in relapse (37.7%) or were refractory to intensive (37.5%) or nonintensive (43.2%) therapies [62]. Phase III trial on the enasidenib is still under process, it will become a potent lead entity for anticancer treatment in the future.

## TARGETED CRITICAL SIGNALING PATHWAY IN R/R AML

### Bcl-2 inhibitor

Venetoclax. Venetoclax (VEN) is highly selective, oral small-molecule B cell leukemia/lymphoma-2 (BCL2) inhibitor. In patients with relapsed and refractory (R/R) AML, VEN had a modest single-agent activity (19% CR/CRi) [63]. In contrast, VEN in combination with HMAs demonstrated significant activity in R/R AML. In a retrospective study, 33 r/r AML patients were treated with HMA plus VEN, the results showed that the ORR was 64%, and 1-year OS was 53% [64]. In a multicenter historical study, 23 R/R AML patients were treated with a combination of VEN and HMA. About 43% achieved a CR or CRi. Median OS was 5.6 months. Median OS for patients achieving CR was longer when compared with patients achieving Cri (10.8 months, 95% CI 6.2-15.4 vs. 2.8 months, 95% CI 0.9-4.8, *p* < 0 .001) and the 6-month projected OS was 80% versus 12% [65]. Another retrospective study of 43 R/R myeloid patients treated with VEN plus HMA or low-dose cytarabine (LDAC), observing objective response of 21%, and median survival of 3 months [66]. A retrospective study of 14 R/R AML was performed to evaluate the efficacy of VEN combination therapy, obtaining an objective response rate of 35.7% and mOS of 4.7 months. There was no difference in response if prior stem cell transplant or HMA exposure [67]. The overall CR/CRi rate with VEN-HMA was 42% in r/r AML in Aldoss’s study [68]. In a phase 2 trial, 55 R/R AML received VEN with 10-day Dec therapy, reaching an ORR of 62%. The mOS was 7.8 months in R/R AML group [69]. In a retrospective study, 40 R/R AML patients received VEN-based therapy, obtaining an ORR of 50% and CRR of 22.5%. Median time to best response was 1.4 months and the mOS was 6.6 months. Patients in intermediate-risk cytogenetics demonstrated better OS than unfavorable-risk cytogenetics [70]. Therefore, combination therapy consisting of venetoclax and HMA is promising in patients with R/R AML. In addition, VEN-based therapy is also a potent therapy option for patients relapsing after HCT. A recent study reported the outcomes of 21 post-HCT AML relapse patients treated with VEN. Of the 19 patients who were assessed for response, VEN yielded an ORR of 42.1%. About 47.4% patients maintained their response for ≥3 months and 8 patients were still receiving therapy at time cut [71]. The objective response rate of VEN-based combination therapy was raging from 20% to 70% in R/R AML patients. It is urgent to conduct larger prospective and randomized clinical trials to evaluate novel venetoclax-based combination chemotherapy fit for R/R AML patients. In summary, venetoclax especially in combination therapy is promising in R/R AML.

Drug–drug interactions with moderate to strong CYP3A4 inhibitors, which are “azole” antifungals, are an important factor to considered for venetoclax plus HMA clinical trials, and these therapies were not permitted in most patients [14,72]. CYP3A4 inhibitors can increase the serum drug concentration of VEN. Agarwal et al.’s study found that compared with monotherapy of VEN, coadministration of oral posaconazole increased venetoclax dose–normalized Cmax and AUC0–24 7.1- and 8.8-fold, respectively. Posaconazole can be used for antifungal prophylaxis in AML receiving VEN after reducing the VEN dose by at least 75% [73]. In patients treated with VEN plus HMA without azole prophylaxis, the rate of grade 3/4 fungal infections was 8%, while 46% of patients received non-azole antifungal (such as echinocandin) prophylaxis [14]. The routine use of antifungal prophylaxis is not recommended in the treatment of AML, and the clinical efficacy of the reduced dosed of VEN when coadministration with azoles is uncertain, antifungal prophylaxis with VEN plus HMAs needs not be mandatory [74]. However, when AML patients with neutropenic occurred, aggressive antifungal treatment therapy is necessary. Echinocandins can be effective antifungal therapies which do not require venetoclax dose reductions. However, it may be limited by the efficacy, delivery mechanisms, and costs. Therefore, azole antifungals will be a better choice at that setting. For strong CYP3A4 inhibitors, the recommended VEN dose reduction from 400 mg is 70 mg for posaconazole and 100 mg for other strong inhibitors (such as voriconazole). For the moderate CYP3A4 inhibitor isavucaonzole, it is recommended to decrease the venetoclax dose to 200 mg [74]. Unlike azoles, antiviral and antibacterial prophylactic agents do not require venetoclax dose adjustments. In addition, except grade 3-4 hematological toxicity, the most common side effects including hypocalcemia (16%-87%), hyperglycemia (67%), hyperkalemia (17% to 59%), increased serum aspartate aminotransferase (53%), decreased serum albumin (49%), hypophosphatemia (45%), diarrhea (43%), nausea (42%), hyponatremia (40%), upper respiratory tract infection (36%), fatigue (32%), musculoskeletal pain (29%), hyperphosphatemia (14%), abdominal pain (18%), constipation (16%), vomiting (16%), mucositis (13%), and tumor lysis syndrome (2-3 weeks promotion stage: 13%; 5-week promotion stage: 2%). Therefore, it is recommended to monitor liver and kidney function and electrolytes throughout VEN therapy.

### Hedgehog pathway inhibitor

#### Glasdegib

Glasdegib is the hedgehog pathway inhibitor. Glasdegib in combination with low-dose cytarabine (LDAC) was approved by FDA for treatment of newly diagnosed AML in adults who are ineligible for intensive chemotherapy in November 2018 [75]. For AML patients who are ineligible for intensive chemotherapy, the addition of glasdegib to LDAC demonstrated significant and meaningful OS improvement. In a phase II study, ORR with glasdegib plus LDAC (26.9%) was higher compared with LDAC (5.3%). Furthermore, patients treated with glasdegib plus LDAC achieved a 49% reduction in the risk of death relative to LDAC (median 8.8 vs. 4.9 months; *p* = 0.0004). The most common AEs were cytopenias and gastrointestinal events (mostly grade 1-2) in glasdegib plus LDAC arm [15]. Tavares et al. reported the outcome of 6 patients with R/R AML or HR-MDS treated with glasdegib. Four (66.7%) patients achieved stable disease after 2 months of treatment. Four patients survived more than 6 months, with a median follow-up of 7 months (0.1-15.1 months) [76]. Similar results have been observed with monotherapy of glasdegib in refractory myelodysplastic syndromes (MDS). In a phase 2 trial, 35 patients with HMA-failure MDS were enrolled to evaluate the efficacy and safety of glasdegib. The ORR was 6%, with the best response of marrow complete remission with hematologic improvement. With a median follow-up of 42.8 months, the mOS was 10.4 months. Grade 3 or higher infections occurred in 11% of patients, and non-hematologic toxicities were rare [77]. Further studies including more R/R AML or R/R MDS patients should be conducted to explore the efficacy and safety of combinations of glasdegib with other novel agents or standard approved therapies.

## TARGETED CELL SURFACE ANTIGEN IN R/R AML

### Antibody-drug conjugate (ADC)

ADC is a novel therapy that combines a monoclonal antibody with targeting specificity and a small molecule with high toxicity. CD33 antigen is a pleasant target for R/R AML. CD33 is expressed on more than 90% of AML patients, while expressed not on pluripotent hematopoietic stem cells, thus avoiding permanent inhibition of the hematopoietic system. Gemtuzumab ozogamicin (GO) is an ADC composed of an anti-CD33 monoclonal antibody covalently linked to the DNA-cleaving cytotoxic agent calicheamicin. The phase III study showed that monotherapy of GO can prolong the OS and recurrence-free survival of newly-treated or R/R AML patients who cannot tolerate standard chemotherapy [78]. In R/R AML, monotherapy with GO has shown a 26-33% OR rate, with a mOS of 4-6 months, but with high degree of hematological and liver toxicities [79-81] GO is generally well tolerated in patients with R/R AML or APL, but single agent treatment may show higher adverse reaction rate. In a recent study, 331 patients received GO as monotherapy for R/R AML (n = 139), combination therapy for R/R AML (n = 183), or treatment for R/R APL (n = 9). Corresponding treatment discontinuations occurred in 68, 39, and 33% of patients. All-causality grade 5 AEs occurred in 52, 22, and 22% of patients in the monotherapy, combination, and APL groups, respectively. Corresponding grades 3 and 4 treatment-related AEs were reported in 60, 55, and 78% of patients. Hepatotoxicity occurred in five patients: Veno-occlusive disease (n = 4) and drug-induced liver injury (n = 1) [82]. However, compared with single agent, the addition of cytarabine and mitoxantrone to GO has presented a higher ORR (60-70%) and longer survival (mOS more than 9 months) [83]. A GO-based intensive regimen can be a viable option for salvage therapy and as a bridge to allogeneic transplant. In a French study of 58 primary refractory or relapsed acute myeloid leukemia (AML) patients with a median age at salvage of 56 years, the combination of fractionated GO with cytarabine and mitoxantrone achieved an ORR of 67%, and the leukemia-free survival (LFS) and OS at 2 years was 36% and 54%, respectively. Incidences of nonrelapse mortality, grade II-IV acute graft-versus-host disease (GVHD) and chronic GVHD were 16%, 40%, and 45%, respectively [84].

### Bispecific antibody

Not only ADCs but also bifunctional antibodies are under investigation. The results of a phase I clinical trial showed that the CR/CRi rate of CD123 × CD3 bifunctional antibody (Flotetuzumab) in patients with relapsed AML after chemotherapy was 31%, but no treatment response was observed in patients with refractory AML [85,86]. The tolerability and anti-leukemia activity of the CD33×CD3 bifunctional antibody AMG330 in R/R AML patients have also been confirmed [87]. More bispecific antibodies such as SGN-CD33A (CD33 antibody-coupled drugs), IMGN779 (CD33 antibody-coupled drugs), and IMGN632 (CD123 antibody-coupled drugs) are under study [88].

### Hypomethylating agents

The demethylating drugs decitabine or azacitidine have shown encouraging effects in the treatment of R/R AML, especially in elderly patients who are unsuitable for intensive chemotherapy or transplantation. Due to the different conditions of the enrolled patients, the ORR of HMA combined therapy is about 30%-60% [9-12,14,25-27,36]. A recent phase III trial of oral Aza (CC-486) as maintenance therapy in patients with AML who are in first remission after intensive therapy showed that CC-486 maintenance therapy prolonged the overall and relapse-free survival compared with placebo (24.7 months vs. 14.8 months, 10.2 months vs. 4.8 months, respectively). The most common AEs were grade 1 or 2 gastrointestinal symptoms. Common grade 3 or 4 AEs were neutropenia and thrombocytopenia [89].

## DISCUSSION

Targeted agents provide new options for the treatment of R/R/AML and obtain a certain remission rate. However, the median duration time of CR is short, and the prolongation of patient OS is not ideal. The results of targeted agents for R/R AML/MDS are listed in Table 1. Results of several clinical trials confirmed that application of targeted agents in earlier time can obtain a deeper and more sustainable remission, thus get survival benefits for R/R AML patients. VEN combined with HMAs for naive and elderly AML patients obtain a complete remission of 73%, with a median survival time of 17.5 months [14]. In patients with R/R AML, VEN combined with HMAs obtained an ORR of <60%, while the mOS decreased for <6 months [65-69]. Therefore, the best time to choose targeted agents needs further exploration.

More than one driver mutation participates in the pathogenesis of AML. The genomic and epigenomic landscapes of AML reported 13 coding mutations in genes, with an average of 5 of these are recurrently mutated in AML [13]. A higher genetic complexity was observed in R/R AML. At the time of relapse, genomic alterations significant increased [90]. Previous study compared the mutation analysis of primary and relapse samples, and the results revealed a high stability for mutations in DNMT3A, IDH2, and NPM1, whereas FLT3-ITD and IDH1 were less stable. However, the majority of FLT3-TKD and NRAS mutations presented in the primary leukemia were loss at relapse. At relapse, FLT3-ITD mutations are acquired in 33% of FLT3-ITD negative patients. In addition, most aberrations were exclusively found in relapse samples as opposed to a diagnosis, suggesting that aberrations in R/R AML were induced by chemotherapy or were present in small subclones selected by therapy [90]. Therefore, multiple new critical abnormalities may be obtained in the evolution of R/R AML, and then these abnormalities will be an active driver in the disease progression. Targeted agents generally only target specific targets, but in the course of disease progression, factors such as clonal evolution, genes loss or recurrent, and frequent emergence of functionally heterogeneous subclones may affect the efficacy of targeted agents. Hence, trials focusing on the isolated testing of novel targeted agents are problematic, and combination therapy may be an important solution under the assumption of a reasonable biological principle. Different agents modulate distinct pathways or targets, and can be administered simultaneously or sequentially [91]. For example, MEK or MDM2 inhibition can down-regulate MCL1 and overcome resistance to BCL-2 inhibition [92]. A Phase I study of BCL2 inhibitor VEN and MDM2 inhibitor idasanutlin in R/R AML was discontinued because of the poor efficacy, the safety was well tolerated, suggesting that the combination of targeted agents was feasible for R/R AML [93].

Tumor immunotherapy has been proven to have continuous immune surveillance. Studies of targeted therapy combined with immunotherapy are ongoing. Preclinical study showed that FLT3-inhibitor can upregulate the surface expression of FLT3 specifically on FLT3-ITD+ AML cells and enhance the recognition of FLT3-CAR T-cells *in vitro* and *in vivo*. This indicates that CAR T-cell immunotherapy in combination with small molecule inhibitor can be used to exert anti-leukemia efficacy [94]. Preclinical models conformed that blocking PD-1/PD-L1 pathways enhanced anti-leukemic responses. A phase II study of Aza plus Nivolumab in R/R AML achieved an ORR of 33%, with 9% of the patients obtaining stable disease over 6 months. Grade 3 to 4 immune-related AEs occurred in 11% of the patients [95].

## CONCLUSION

In summary, the emergency of therapeutic strategies targeting mutated genes, cell surface markers, cell signal transduction pathways, and immune responses making R/R AML treatments gradually diversified and achieved well therapeutic effects. More individualized and precise treatment should be involved for the treatment of R/R AML in the future.
